# Preoperative vocal cord paralysis and its association with malignant thyroid disease and other pathological features

**DOI:** 10.1186/s40463-015-0087-1

**Published:** 2015-09-11

**Authors:** Emily Kay-Rivest, Elliot Mitmaker, Richard J. Payne, Michael P. Hier, Alex M. Mlynarek, Jonathan Young, Véronique-Isabelle Forest

**Affiliations:** Department of Otolaryngology – Head and Neck surgery, McGill University, Montreal, QC Canada; Department of General surgery, McGill University, Montreal, QC Canada; Division of Head and Neck Surgery, Department of Otolaryngology – Head and Neck surgery, Jewish General Hospital, McGill University, Montreal, QC H3T 1E2 Canada

## Abstract

**Background:**

Vocal cord paralysis (VCP) is found in both benign and malignant thyroid disease. This study was performed to determine if the presence of preoperative VCP predicts malignancy.

**Methods:**

A retrospective analysis was performed on a cohort of 1923 consecutive patients undergoing thyroid surgery. The incidence of preoperative VCP was recorded. Patient and nodule characteristics were correlated with final pathology.

**Results:**

1.3 % of our cohort was found to have preoperative VCP. Malignant pathology was discovered in 76 % of patients with preoperative VCP. Among these patients, 72 % had a left sided paralysis. 10.5 % of patients with preoperative VCP had perineural invasion (PNI) on final pathology, compared to 1.1 % of patients with normal VC function.

**Conclusion:**

Preoperative VCP appears to be a strong, though not an absolute, indicator of malignancy. Most VCP were on the left side. Assessing for preoperative VCP is crucial in all patients who need thyroid surgery, as even benign nodules can be accompanied by preoperative vocal cord paralysis.

## Introduction

In the past, preoperative vocal cord paralysis was thought to be an absolute marker of thyroid malignancy [1]. However, there are several reports in the literature indicating that benign thyroid disease may also lead to vocal cord paralysis [2–5]. The mechanisms that cause vocal cord paralysis are multiple. In malignant disease, recurrent laryngeal nerve (RLN) paralysis can be caused by direct invasion of malignant cells or by nodular compression. In the case of benign thyroid disease, compression, as well as stretching of the nerve, are possible etiologies. Furthermore, idiopathic VCP unrelated to a thyroid process could also be a cause of a preoperative VCP. The importance of preoperative assessment of the vocal cords is being increasingly recognized since the recent publication of the 2013 American Academy of Otolaryngology—Head and Neck surgery guidelines, which highlighted the importance of this evaluation, even for patients without subjective voice complaints. These guidelines note that at the present time, vocal cord assessment is performed in 6.1 to 54 % of patients in the United States prior to surgery. The objective of this study was to evaluate the presence of vocal cord paralysis before thyroid surgery and to better understand its association with malignant thyroid disease.

## Methods

The health records of 1923 patients who had preoperative flexible laryngoscopy and underwent thyroid surgery between 2007 and 2014 at the McGill University teaching hospitals were analyzed retrospectively. Every patient undergoing thyroidectomy has undergone pre-operative flexible laryngoscopy in our database, unless they came in with an emergent airway obstruction. We recorded all cases of preoperative vocal cord paralysis and correlated with final pathology. The study recorded the sex, age and side of paralysis among patients with confirmed preoperative vocal cord paralysis and final pathology findings; this data was available for all patients. In addition, the size of the largest nodule seen on ultrasound was also recorded, if documented. Furthermore, histopathological subtypes of thyroid cancer, including the presence of extrathyroidal extension (ETE), perineural invasion (PNI), and lymphovascular invasion (LVI) were examined with respect to the presence or absence of preoperative vocal cord paralysis. Data was analyzed using MedCalc 12.0. Chi-square and student’s t-test were used to identify differences between groups. A *p-value* of 0.05 or less was considered statistically significant. Ethical review board approval was obtained from our institution (the Jewish General Hospital) for this study. None of the authors have any competing interests in the manuscript.

## Results

Preoperative vocal cord paralysis was diagnosed in 25 out of 1923 patients (1.3 %). Within the vocal cord paralysis group, 72 % of patients were women. In the group without VCP, 77 % of patients were women (*p* = 0.3644). The average age of patients with VCP was 59.9 years, compared to 50.7 years in patients without preoperative VCP (*p* = 0.0016). These results are summarized in Table 1.

**Table 1:** Demographics of all patients who underwent thyroid surgery from 2007 to 2014

		Preoperative vocal cord paralysis		
		Normal mobility	Paralysis	
Total		1898 (98.7 %)	25 (1.3 %)	
Age	<30	139 (7.3 %)	0 (0 %)	
	30–39	285 (15.0 %)	1 (4.0 %)	
	40–49	480 (25.2 %)	5 (20.0 %)	
	50–59	470 (24.7 %)	5 (20.0 %)	
	60–69	327 (17.2 %)	7 (28.0 %)	
	70–79	152 (8.0 %)	3 (12.0 %)	
	>80	44 (2.3 %)	4 (16.0 %)	
				*p-value*
Mean; range of age (years)		50.7; 15–94	59.9; 36–88	0.0016
Sex	Male	424 (22.35 %)	7 (28 %)	0.3644
	Female	1474 (77.7 %)	18 (72 %)	

Upon further analysis of the 25 patients with confirmed preoperative vocal cord paralysis, we found a left cord paralysis in 72 % of cases. Furthermore, 19 patients (76 %) had malignant final pathologies and 6 had benign nodules. Twelve (48 %) out of the 19 patients with cancer were papillary carcinomas, 4 (16 %) were micropapillary carcinomas, one was a medullary carcinoma, one was a poorly differentiated thyroid carcinoma and one was an osteosarcoma. The benign thyroid pathologies consisted of thyroid follicular adenomas (*n* = 2) and nodular hyperplasia (*n* = 4) (Table 2). In the cohort of patients with normal preoperative vocal cord movement, 1143 (60.2 %) patients had malignant final pathologies (*p* = 0.2218) (Table 3).

**Table 2:** Demographics and pathology results of 25 patients with preoperative vocal cord Paralysis

Subject	Sex	Side	Largest nodule on U/S (cm) and side	Final pathology	Adverse pathological features
1	F	R	2.4 (right)	Medullary CA	
2	M	L	1 (left)	Micropapillary CA	ETE
3	M	L	1.2 (left)	Micropapillary CA	
4	M	L	1.2 (left)	Papillary CA	
5	F	R	1.4 (right)	Follicular adenoma	
6	M	R	1.6 (left)	Papillary CA	
7	F	L	1.9 (left)	Nodular hyperplasia	
8	F	L	2.15 (left)	Papillary CA	
9	F	R	2.3 (right)	Papillary CA	
10	F	L	2.4 (midline)	Follicular adenoma	
11	F	L	2.7 (left)	Micropapillary CA	
12	F	L	3 (left)	Papillary CA	
13	F	L	1.7 (left)	Papillary CA	
14	F	L	3.5 (left)	Nodular hyperplasia	
15	F	R	Irregular, >3^a^	Papillary CA	ETE, PNI, LVI
16	M	L	5.5 (left)	Papillary CA	
17	F	L	6.5 (left)	Nodular hyperplasia	
18	F	R	7.7^a^(right)	Papillary CA	
19	M	L	9.1 (left)	Papillary CA	ETE, LVI
20	F	L	7 (left)	Nodular hyperplasia	
21	M	L	0.7 (left)	Micropapillary CA	
22	F	L	No imaging^c^	Osteosarcoma^b^	
23	F	R	1.5 (right)	Papillary CA	
24	F	L	No imaging^c^	Poorly differentiated CA	
25	F	L	No imaging^c^	Papillary (tall cell variant) CA	ETE, PNI, LVI

(*M* male, *F* female, *U/S* ultrasound, *CA* carcinoma, *ETE* extrathyroidal extension, *PNI* perineural invasion, *LVI* lymphovascular invasion)

^a^Measure obtained from a CT Scan. No U/S available for this patient

^b^See discussion section regarding this finding

^c^These patients did not have preoperative imaging as they arrived in respiratory distress

**Table 3:** Status of preoperative vocal cord function according to pathology results

	Preoperative vocal cord paralysis n (% of total)		*p-value*
	+	-	
Malignant pathology	19 (1.0 %)	1143 (59.6 %)	0.2218
Benign pathology	6 (0.3 %)	671 (35.0 %)	
Total	25 (1.3 %)	1814 (98.7 %)	

When ultrasound results were available, we used the largest-sized nodule on the side of the paralysis for patients with preoperative VCP, and the largest nodule overall in patients without VCP. Patients with malignant final pathology had a mean nodule size of 2.9 cm and those with benign final pathology had an average nodule size of 3.2 cm (*p* = 0.0001). Regardless of pathology results, the mean size of nodule in patients with normal vocal cord function was 3.0 cm (range of 0.3–13.0 cm) and it was 3.3 cm (0.7–9.1 cm) in patients with preoperative VCP (*p* = 0.5916). Malignant nodules found in patients with normal vocal cords (mean = 2.89 cm) were smaller than the malignant nodules of patients with preoperative VCP (mean = 3.2 cm), but it was not statistically significant (*p* = 0.4168). On the other hand, benign nodules of patients with normal vocal cords (mean = 3.22 cm) were larger than benign nodules found in patients with preoperative VCP (mean = 3.14 cm). However, this finding was also not statistically significant (*p* = 0.9651).

Other known adverse pathological features, such as ETE, LVI and PNI were also examined. Among patients with preoperative VCP, 21.1 % were found to have ETE, whereas 13.2 % of patients with normal vocal cord function had ETE on pathology. This finding was not statistically significant (*p* = 0.27). For LVI, results were comparable between patients with and without preoperative VCP (15.8 % in the VCP group and 10.8 % for patients with normal VC, *p* = 0.4837). However, PNI was significantly more common in patients with preoperative VCP (10.5 %) compared to patients without VCP (1.1 %) (*p* = 0.0028). These results are summarized in Table 4.

**Table 4:** Comparison of the incidence of adverse pathological features found in thyroid cancers of patients with and without preoperative VCP

Patients with malignant pathology			
	Without VCP	With preop VCP	*p*-value
Perineural invasion	13 (1.13 %)	2 (10.5 %)	0.0028
Lymphovascular invasion	123 (10.76 %)	3 (15.78 %)	0.4837
Extrathyroidal extension	151 (13.21 %)	4 (21.05 %)	0.2700

## Discussion

Vocal cord paralysis can occur in both benign and malignant thyroid disease. The etiology of the paralysis is not always evident, but can often be explained by direct invasion or compression of the recurrent laryngeal nerve by malignant thyroid disease. In the case of benign thyroid disease, the paralysis could be idiopathic or could be caused by compression or stretching of the nerve. Knowledge of preoperative vocal cord paralysis is imperative for appropriate surgical planning and management. In this study, 1.3 % of patients undergoing thyroid surgery were found to have preoperative VCP. The true incidence of VCP in the general population is difficult to ascertain. A study by *Shafkat et al.* looked at 30,262 patients who attended an ENT outpatient clinic during a 2 year period [5]. One hundred ten of these patients were diagnosed with vocal cord paralysis, an incidence of 42 per 10,000 new patients [5]. Several studies have evaluated all causes of VCP and have found them to be idiopathic in 16.3 % [6], 17.4 % [7], 17.6 % [8] and 31.1 % [9] of patients.

Although the majority of patients undergoing thyroidectomy were women, there was no significant difference between men and women in terms of the presence or absence of preoperative VCP. On the other hand, our study found age to be a significant factor; it was considerably higher in patients with preoperative VCP. *Chiang et al.* also noted this finding in a study looking at thyroid tumours with preoperative recurrent laryngeal nerve palsies [10]. One explanation for this result would be that nodules have more time to evolve and develop adverse features with advancing age.

In our cohort, 76 % of patients with preoperative vocal cord paralysis were found to have malignant disease. Several other studies have also shown preoperative vocal cord paralysis to be a robust marker of malignancy [2–5]. However, it is not a perfect marker. Indeed, in our cohort, 24 % of patients with vocal cord paralysis had benign final pathology results. This finding emphasizes the importance of evaluating the vocal cords of all patients scheduled for thyroid surgery, regardless of fine-needle aspiration (FNA) results.

Many techniques are used to assess vocal cord function preoperatively. Presently, the flexible laryngoscope remains the most widely used. Another promising technique to study the vocal cords is transcutaneous laryngeal ultrasound. *Wang et al.* reported that it accurately diagnosed both normal and palsied vocal folds in 90 % of female patients, with a lesser success rate in males over 40 years of age [11]. The rate of false negatives with this technique appears to be one reason why it has not been widely adopted in the adult population [12–13].

The literature shows a preponderance of left-sided paralysis, and data regarding this finding dates back to the 1940’s [15]. According to *Meurman et al.*, the left recurrent laryngeal nerve is more likely to be injured because it branches off the vagus nerve more caudally on the left side as compared to the right. In point of fact, after branching off the vagus nerve, the left recurrent laryngeal nerve, unlike the right, loops under the aortic arch. It then runs upward in the groove between the trachea and esophagus with a more medial course, closer to the thyroid gland than to the right recurrent laryngeal nerve. Our results show that 72 % of patients had a left-sided paralysis, a finding similar to other reported studies by *Benninger et al., Terris et al.* and *Pavithran et al*. Another reason for the preponderance of left-sided paralysis may lie in the length of each nerve [14–16]. On average, the left recurrent laryngeal nerve measures 12 cm, whereas the right one measures 5 to 6 cm [17]. The added length may explain the higher rate of left-sided paralysis, providing more regions of vulnerability along the nerve [17].

We studied the relationship between nodule size and malignancy. The only statistically significant finding was that overall, benign nodules were slightly larger than malignant ones, regardless of the status of vocal cord function. We found no significant correlation between the size of the nodules and the function of the vocal cords. Indeed, some of the largest nodules corresponded to benign pathologies in both VCP and no VCP groups. This finding is consistent with the literature [18–21]. In 2011, *Moon et al.* published a consensus statement and recommendations on ultrasonography and the ultrasound-based management of thyroid nodules [21], concluding that thyroid nodule size was not helpful for distinguishing malignant and benign nodules. Furthermore, *Kamran et al.* reported that above 2 cm, there was a non-linear relationship between nodule size and cancer risk [18]. It is hypothesized that other mechanisms, such as stretching or compression of the recurrent laryngeal nerve, could be the cause of paralysis. However, other reports suggest that increasing size may indeed correspond to a greater risk of malignancy. For example, *Raparia et al.* described a statistically significant increased risk of malignancy in nodules over 2 cm in size [22].

Histopathological tumor characteristics provide crucial information when it comes to prognosis and risk of recurrence. When these characteristics were retrospectively analyzed in our study, in the setting of preoperative VCP, we found perineural invasion to be far more common in patients with preoperative VCP. No such association was found with extrathyroidal extension and lymphovascular invasion. According to our results, when a preoperative VCP is present, a patient had a 76 % chance of harbouring a thyroid malignancy, with a higher chance of finding perineural invasion on final pathology. With this knowledge, the degree of surgical aggressiveness can be planned accordingly.

There are limitations to this study. First, it is a retrospective analysis. Second, despite a large sample size, the incidence of preoperative VCP is very low overall. Therefore, evaluation of the clinical characteristics of thyroid pathologies remains limited.

## Conclusion

This study demonstrates that preoperative vocal cord paralysis is a robust marker of malignancy risk, although not an absolute one. It most commonly affects the left vocal cord. It also emphasizes the importance that every patient undergoing thyroid surgery should have a preoperative vocal cord assessment, even in the context of a benign FNA result, as 24 % of patients with preoperative VCP in this study had benign thyroid pathologies, as demonstrated in several other studies [1, 3–5, 23–30].
